# Histological and Clinical Features of Primary and Recurrent Periocular Basal Cell Carcinoma

**DOI:** 10.5402/2012/354829

**Published:** 2012-04-24

**Authors:** Guy J. Ben Simon, Semion Lukovetsky, Fabio Lavinsky, Nahum Rosen, Mordechai Rosner

**Affiliations:** Goldschleger Eye Research Institute, Tel-Aviv University, Sackler School of Medicine, Sheba Medical Center, Tel-Hashomer 52621, Israel

## Abstract

*Background*. Basal cell carcinoma (BCC) is the most common malignancy of the eyelids. Medial canthal BCCs tends to recur more often. *Purpose*. To evaluate the clinical and histological features of primary and recurrent periocular BCC, in order to identify any existing associations. *Methods*. Data from 87 patients (71 primary and 16 recurrent) were analyzed in this study. All patients underwent tumor excision with frozen section margin control at the Goldschleger Eye Institute between 1/1995 to 12/1997. Statistical analysis was performed to identify possible associations between histological and clinical characteristics of primary and recurrent BCC. *Main Outcome Measures*. Anatomical location, clinical presentation, and histology of peri-ocular BCC. *Results*. No association was found between histopathological and clinical characteristics of BCC. Similar features with regard to eyelid location and histology were found in primary and recurrent peri-ocular BCCs, whereas recurrent BCCs tended to involve a greater eyelid extent with a longer duration of symptoms. Medial canthal BCCs, morpheaform, or sclerosing histology were not more common in the recurrent BCC group. *Conclusions*. Similar clinical and histological characteristics were noted in primary and recurrent periocular BCC, implying that incomplete surgical excision rather than anatomical location or histological features is the main cause for recurrence.

## 1. Introduction

 Basal cell carcinoma (BCC) is the most common skin cancer and accounts for 80–95% of all eyelid tumors. Prolonged exposure to sunlight seems to be an important predisposing factor [1], explaining why the tumor occurs more frequently on the lower eyelid [2]. BCC originates as a neoplastic transformation of the basal cells of the epidermis that proliferate and invade into the dermis as bulbous nodules or invasive strands. Classification can be made according to its clinical features, histological differentiation, or growth patterns. While several clinical types have been noted, there are multiple histopathological differentiations of BCC [3, 4].

BCC generally exhibits slow growth. The tumor may invade the adjacent tissues and cause their destruction but it rarely metastasizes [2, 5]. The goal of treatment is to completely excise the BCC in order to prevent recurrence. While several treatment modalities have been described, complete excision with margin control either by frozen sections or Mohs' micrographic surgery (MMS) remains the gold standard and assures low recurrence rate, with incomplete excision being the main cause for recurrence [5–11]. Several studies suggest that specific location such as medial canthus and certain histological differentiation such as morpheaform or sclerosing BCC may be associated with higher recurrence rates [5–11].

 In the current research, an attempt was made to identify correlations between histopathological features and various clinical characteristics of BCC, such as tumor location, size, and histology type. In addition, the study investigated whether differences exist between primary and recurrent BCC, with respect to various clinical and histological features. No differences found between these two groups of BCC would lend further support to the assumption that the main cause for recurrence is incomplete excision.

## 2. Methods

In this retrospective study, all patients underwent BCC excision at the Goldschleger Eye Institute during a 3-year period (January 1995 to December 1997).

Data regarding patients' demographics, tumor location, tumor size, and histological features were collected and analyzed. All lesions involving the canthal area with or without spreading to the eyelids were classified as canthal lesions.

The BCC was classified into 3 groups according to macroscopically measured tumor size: small, <10 mm in its longest axis; medium, 10 to 20 mm in its longest axis, and large, >20 mm in its longest axis. Histological patterns of BCC were determined and when solid and infiltrative patterns were found in the same tumor it was classified as mixed type.

### 2.1. Surgical Technique

Surgery was performed under local anaesthesia. The first stage involved resection of the macroscopically visible tumor with clinically free margins of 1 mm. The specimen was fixed in formalin for further histopathology. Margins of 1 mm from the remaining tumor bed were then excised, and frozen sections of these tissue strips were evaluated. The results of the microscopic examination were reported to the operating theatre before completion of surgery. If margins were not free of tumor, additional tissue excision from the involved side was performed, and the frozen sections were examined again for tumor presence. Only when all margins were reported to be free of tumor residue was the surgical wound closed and the surgery completed.

### 2.2. Histopathological Examination

After fixation in formalin, the main tissue specimen was measured and sectioned into three pieces that included the various margins of the lesion. These tissue sections were then embedded in paraffin. Sections of 4 *μ*m were prepared from the paraffin block using a microtome, mounted on glass slides and stained by haematoxylin and eosin for microscopic evaluation.

All lesions were reevaluated and classified histologically based on the classification described by Wade and Ackerman [3]. The following 9 types of BCC were recognized: solid, nodular, nodular-ulcerative, morpheaform, infiltrative, cystic, nodular-cystic, basosquamous, and plexiform (adenoid pattern).

Institutional review board (IRB) was waived for the study.

### 2.3. Statistical Analysis

The Wilcoxon Mann-Whitney test and chi-square nonparametric analyses were used to compare numerical variables and proportions, respectively, between primary and recurrent BCCs. The Fisher's Exact test was used to examine the probability of a lesion to occur in a specific anatomical location. Statistical analysis was carried out using Microsoft Excel 2003 (Microsoft Corporation, Redmond, WA) and SPSS version 13.0 (SPSS, Inc., Chicago, IL) programs.

## 3. Results

Eighty-seven patients (52 males, 35 females, mean age of 70 years, range 33–96 years) participated in the study. Average symptom duration was 34 months (range 1 month to 30 years), and average time to surgery was 2.4 months. The left eye was more commonly affected, with 58% of lesions appearing on this side. Lesion size was 40% of eyelid extent on average. Most lesions appeared on the lower eyelid (55 lesions, 63%), followed by medial canthus (13 lesions, 15%), upper eyelid (12 lesions, 14%), and lateral canthus (7 lesions, 8%). Seventy-one lesions (82%) were primary BCC and 16 lesions were recurrences (18%) that were referred from different hospitals.

### 3.1. Histological Features

Most lesions involved the dermis and orbicularis (65 lesions, 77%), 16 lesions involved the tarsus or conjunctiva (19%), and only 3 lesions (3%) had orbital involvement. No data regarding depth of invasion were obtained from 3 cases.

**Figure 1:**
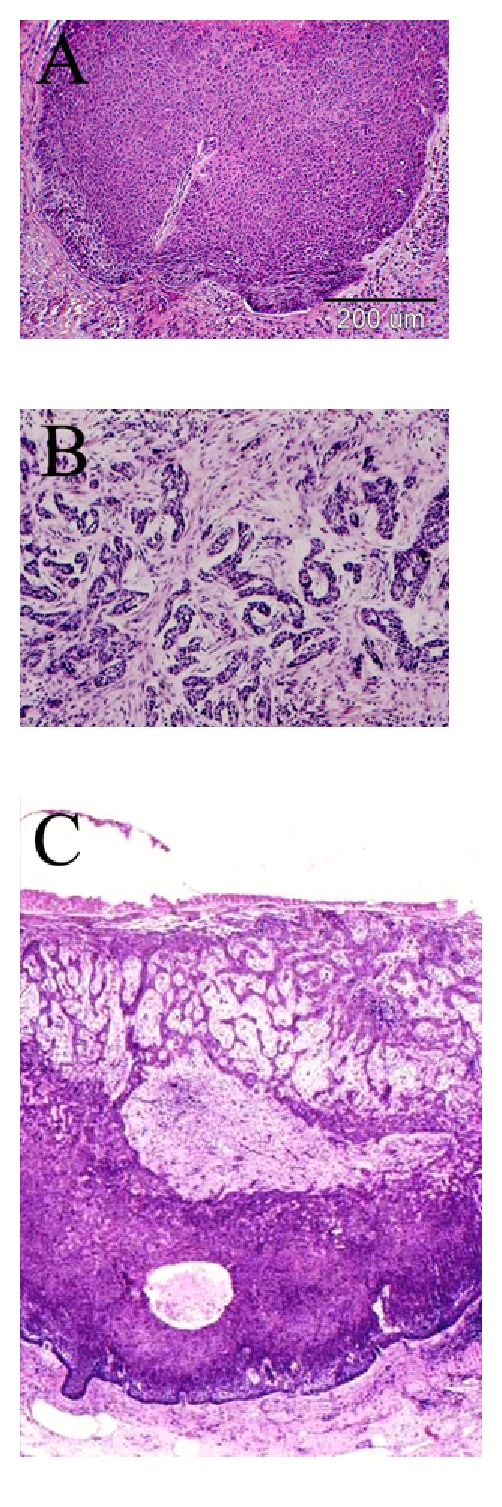
Histology composite of three types of basal cell carcinoma (Haematoxylin and Eosin stain). (A) Nodular type, (B) infiltrative (morpheaform), and (C) mixed.

**Figure 2:**
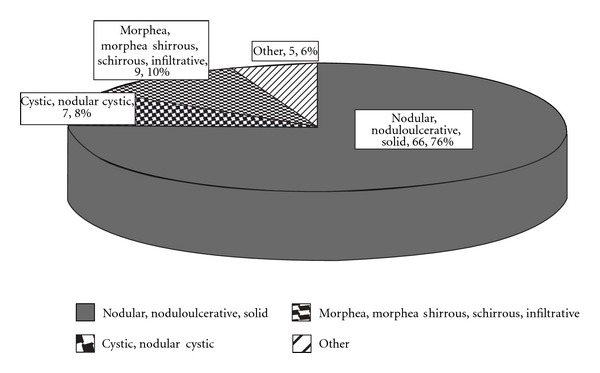
Histological types of 71 patients with primary periocular basal cell carcinoma (BCC) and 16 patients with recurrent BCCs.

Most lesions were solid, nodular, or nodular ulcerative (66 lesions, 75.9%), followed by morphea or infiltrative (9 lesions, 10%) and cystic or nodular cystic (7 lesions, 8%). Other histological forms appeared less commonly and included basosquamous and plexiform (adenoid pattern) (Figures 1 and 2).

Average inflammation score (on an arbitrary scale of 1–3) was 2.1 while spaces in lesions (on a similar scale) was 1.5. Necrosis appeared in 22 cases (25%).

#### 3.1.1. Recurrent versus Primary BCC (Table 1)

Recurrent BCCs were significantly larger than primary lesions (recurrent BCCs involved 46% of eyelid extent versus 36% of eyelid extent in primary lesions, *P* = 0.009, Wilcoxon Mann-Whitney). Duration of symptoms was significantly longer in the recurrent group (52 versus 29 months, *P* = 0.027, Wilcoxon Mann-Whitney test). In addition, recurrent lesions tend to involve the orbit more commonly (*P* = 0.006, Chi-square).

However, no differences between recurrent and primary BCCs were found with respect to age at presentation, eyelid location, and histological differentiation (Table 1).

## 4. Discussion

In the current study, similar clinical and histological features were found in patients with primary and recurrent BCC of the eyelid. No differences in tumor location or histology type were found between the two groups of patients, suggesting that incomplete tumor excision may be the major cause of lesion recurrence.

In our study, the left periocular region was found to be involved more commonly than the right side. This may reflect greater sun exposure on that side during driving, in agreement with the fact that in Australia, where drivers sit on the right side, involvement of the right peri-ocular region is more common than the left [12]. In our study, the lower eyelid was the most common anatomical site of BCC, followed by medial canthus and upper eyelid, in agreement with previous reports [12–14]. Similarly, nodular BCC was the most common histological type followed by morpheaform or infiltrative BCC.

The fact that recurrent lesions tend to be of longer duration and to involve a greater extent of the eyelid is somewhat surprising, since patients who have already undergone eyelid surgery for the primary lesion should be more aware of the possibility of recurrence. This observation was noted in a previous study regarding recurrent lesions that were larger with more subclinical extension [14].

Recurrence after incomplete excision is reported to be 20% with 5 to 9 years of followup [15, 16]. In our group, 16.4% of lesions were recurrent. However, as the patients had their primary surgery in other institutions, the surgical details for primary lesions were not always available.

Similar histological differentiations and eyelid location were found in both groups of primary and recurrent lesions, in contrast to many studies reporting certain features more common in recurrent BCCs [17, 18].

Incomplete excision of BCC is associated with tumor location on the face or inner canthus as well as with infiltrative and multifocal histology types with a reported rate of 10% [17]. Several studies report that medial canthal lesions and morpheaform histology are associated with incomplete resection rate and a significantly higher recurrence rate [15, 16] with incomplete excision being the main risk factor for recurrence. Interestingly, several investigators associate MMS with lower incomplete excision and recurrence rate [8, 13]. However, in our personal experience, MMS may be associated with severe orbital involvement, especially in peri-ocular adnexa deep lesions (data not published). Additional studies report that excision under frozen section control has a 5-year recurrence rate of 2.1%, similar to that for MMS, while excision without frozen section control has a recurrence rate of 5% [12, 19]. Two recent studies support the fact that very small histological safety margins (0.2–2 mm) are required to prevent recurrence of peri-ocular BCCs [20, 21].

Eyelid location along with basosquamous or morpheaform BCCs are reported to be high-risk tumors with extensive subclinical spread that require more MMS levels for tumor clearance [17]. Other factors associated with recurrence include medial canthal location, in-depth extension, and sclerosing type. An increased recurrence rate of up to 14.7% is reported in second surgery and 50% in third surgery [1].

Limitations of the current study stem from its retrospective design. All recurrent BCCs were referrals and no data were obtained regarding the type of initial excision. Similarly, it was not possible to reevaluate the first histological specimens to examine for BCC residues at the margins of dissection. Referral bias may exist, as small lesions might have been excised without margin control during primary surgery or followed up long enough to examine recurrence and more extensive lesions were preferentially sent to our department. In addition, a relatively small number of patients was analyzed in this study, especially in the recurrent BCC group.

Despite these limitations, our study suggests that primary and recurrent peri-ocular BCCs have similar clinical and histological features including eyelid location and tumor type. Medial canthal location, morpheaform, and/or sclerosing histology were not more common in the recurrent BCC group implying that incomplete surgical excision may be the main cause for recurrence.

## Figures and Tables

**Table 1:** Clinical and histological characteristics of primary and recurrent peri-ocular basal cell carcinoma (BCC).

		Primary BCC	Recurrent BCC	*P*
		*N* = 71	*N* = 16	
Age		70 (±13)	68 (±15)	0.7 (ns)

Duration	(months)	29 (±58)	52 (±65)	0.2 (ns)

Time to surgery	(months)	2.5 (±4.8)	1.9 (±1.8)	0.6 (ns)

Size	(percentage)	36 (±8%)	46 (±15%)	0.009

Eyelid location	Lower	45 (63%)	10 (62%)	
	Medial canthus	8 (11%)	5 (31%)	0.2 (ns)
	Upper	11 (15%)	1 (6%)	
	Lateral canthus	7 (10%)	0 (0%)	

Histology	Nodular	63 (89%)	12 (75%)	
	Baso-squamous	2 (3%)	0 (0%)	0.1 (ns)
	Morphea	6 (8%)	3 (19%)	
	Plexiform	0 (0%)	1 (6%)	

Depth of invasion	Dermis	32 (45%)	5 (31%)	
	Orbicularis	25 (35%)	5 (31%)	
	Tarsus	10 (14%)	1 (6.2%)	0.006
	Conjunctiva	3 (4.2%)	2 (12.5%)	
	Orbit	1 (1.4%)	2 (12.5%)	

## References

[1] Pieh S, Kuchar A, Novak P, Kunstfeld R, Nagel G, Steinkogler FJ (1999). Long term results after surgical basal cell carcinoma excision in the eyelid region. *British Journal of Ophthalmology*.

[2] Cook BE, Bartley GB (2001). Treatment options and future prospects for the management of eyelid malignancies an evidence-based update. *Ophthalmology*.

[3] Wade TR, Ackerman AB (1978). The many faces of basal-cell carcinoma. *Journal of Dermatologic Surgery and Oncology*.

[4] Bonner PK, Bregman DK, Mclean IW, La Piana FG (1996). Mixed type basal cell carcinoma of the eyelids. *Investigative Ophthalmology and Visual Science*.

[5] Riedel KG, Beyer-Machule CK, Albert DM, Jakobiec FA (2000). Basal cell carcinoma. *Principles and Practice of Ophthalmology*.

[6] Lindgren G, Larko O (1997). Long-term follow-up of cryosurgery of basal cell carcinoma of the eyelid. *Journal of the American Academy of Dermatology*.

[7] Choontanom R, Thanos S, Busse H, Stupp T (2007). Treatment of basal cell carcinoma of the eyelids with 5% topical imiquimod: a 3-year follow-up study. *Graefe’s Archive for Clinical and Experimental Ophthalmology*.

[8] Nemet AY, Deckel Y, Martin PA (2006). Management of periocular basal and squamous cell carcinoma: a series of 485 cases. *American Journal of Ophthalmology*.

[9] Tuppurainen K (1995). Cryotherapy for eyelid and periocular basal cell carcinomas: outcome in 166 cases over an 8-year period. *Graefe’s Archive for Clinical and Experimental Ophthalmology*.

[10] Mohs FE (1986). Micrographic surgery for the microscopically controlled excision of eyelid cancers. *Archives of Ophthalmology*.

[11] Bandieramonte G, Lepera P, Moglia D, Bono A, De Vecchi C, Milani F (1997). Laser microsurgery for superficial T1-T2 basal cell carcinoma of the eyelid margins. *Ophthalmology*.

[12] Wong VA, Marshall JA, Whitehead KJ, Williamson RM, Sullivan TJ (2002). Management of periocular basal cell carcinoma with modified en face frozen section controlled excision. *Ophthalmic Plastic and Reconstructive Surgery*.

[13] Malhotra R, Huilgol SC, Huynh NT, Selva D (2004). The Australian Mohs database, part II: periocular basal cell carcinoma outcome at 5-year follow-up. *Ophthalmology*.

[14] Malhotra R, Huilgol SC, Huynh NT, Selva D (2004). The Australian Mohs database, part I: periocular basal cell carcinoma experience over 7 years. *Ophthalmology*.

[15] Rodriguez-Vigil T, Vázquez-López F, Perez-Oliva N (2007). Recurrence rates of primary basal cell carcinoma in facial risk areas treated with curettage and electrodesiccation. *Journal of the American Academy of Dermatology*.

[16] Paavilainen V, Tuominen J, Aho VV, Saari KM (2007). Long-term results after treatment of basal cell carcinoma of the eyelid in Southwestern Finland. *European Journal of Ophthalmology*.

[17] Farhi D, Dupin N, Palangié A, Carlotti A, Avril MF (2007). Incomplete excision of basal cell carcinoma: rate and associated factors among 362 consecutive cases. *Dermatologic Surgery*.

[18] Sonia Batra R, Kelley LC (2002). Predictors of extensive subclinical spread in nonmelanoma skin cancer treated with Mohs micrographic surgery. *Archives of Dermatology*.

[19] Holbach LM, Jünemann A, Muhammad S (1998). Surgical management of periocular basal cell carcinoma using frozen section control and immediate plastic reconstruction—indications and methods. *Klinische Monatsblatter fur Augenheilkunde*.

[20] Auw-Haedrich C, Frick S, Boehringer D, Mittelviefhaus H (2009). Histologic safety margin in basal cell carcinoma of the eyelid. Correlation with recurrence rate. *Ophthalmology*.

[21] Chadha V, Wright M (2009). Small margin excision of periocular basal cell carcinomas. *British Journal of Ophthalmology*.

